# Disparities in neighborhood food environment and cognitive decline among US older adults: a cohort study

**DOI:** 10.1186/s12916-025-04091-1

**Published:** 2025-05-06

**Authors:** Boeun Kim, Roland J. Thorpe, Sarah L. Szanton, Paris B. Adkins-Jackson, Laura J. Samuel

**Affiliations:** 1 College of Nursing, University of Iowa, 50 Newton Road, Room 436, Iowa City, IA 52242 USA; 2 Bloomberg School of Public Health, Johns Hopkins University, 624 N. Broadway, Hampton House 708, Baltimore, MD 21205 USA; 3 School of Nursing, Johns Hopkins University, 525 N. Wolfe Street, Baltimore, MD 21205 USA; 4 School of Medicine, Johns Hopkins University, 301 Mason F. Lord Drive, Suite 2500, Baltimore, MD 21224 USA; 5 Mailman School of Public Health, Columbia University, 722 West 168 Street, New York, NY 10032 USA

**Keywords:** Food environment, Healthy eating, Poverty, Nutrition inequity, Social determinants, Neighborhood

## Abstract

**Background:**

Disparities in neighborhood food environments in the United States, attributed to numerous complex economic, social, and political factors, likely to contribute to disparities in access to healthy food and cognitive function in older adults. However, the role of food environment in cognitive function is not well understood. Accordingly, this study examined the association of residing a low food access and low-income neighborhood with changes in cognitive function among older adults in urban areas.

**Methods:**

This is a cohort study leveraging existing datasets. The 2010 Food Access Research Atlas data was linked to the 2011–2021 National Health and Aging Trends Study (NHATS). A total of 4768 urban-dwelling older adults aged 65 years and older were included in this analysis. Total cognitive function (range: 0–33) was assessed through tests of orientation, executive function, immediate memory, and delayed memory. An unhealthy food environment was defined as residing in census tracts with both low access to healthy food stores and low income. Survey-weighted mixed-effects models were fitted, adjusting for individual- and area-level covariates.

**Results:**

The mean age of participants was 77.1 years (SD = 7.6), and 2779 were women (weighted % = 56.7). A total of 1238 participants (weighted % = 9.9%) were racialized as Black, 365 (weighted % = 9.1%) racialized as Latinx, and 3165 (weighted % = 81.1%) racialized as White. In adjusted models, older urban- and community-dwelling adults living in neighborhoods with low access and low income had faster annual cognitive decline than their peers (*β* = − 0.19; 95% CI = − 0.32, − 0.05).

**Conclusions:**

Living in neighborhoods with both low food access and low income may be a risk factor for accelerated cognitive decline among urban-dwelling older adults and contribute to widening disparities in healthy food access and cognitive decline.

**Supplementary Information:**

The online version contains supplementary material available at 10.1186/s12916-025-04091-1.

## Background

A neighborhood food environment refers to the areas where people acquire and consume food [1, 2], including food stores, food retail establishments, community gardens, and food assistance programs. Disparities in the neighborhood food environments in the United States (US) are attributed to numerous complex economic, social, and political factors [3]. In addition to neoliberal economic policies—characterized by deregulation, privatization, and a profit-driven approach—and changes in food supply chains, demographic shifts occurred in large US cities between the 1970s and 1980s where affluent White households moved from urban areas to outlying suburban areas, leading to the closures of stores in the inner cities and the relocation of grocery stores in the suburban [4]. Ongoing disinvestment related to residential segregation or redlining (i.e., historic discriminatory housing practice) in low-income communities lacking purchasing power has restricted food access, which is exacerbated in neighborhoods with residents racialized as Black and Latinx [4–6]. Consequently, low-income areas, such as neighborhoods with residents racialized as Black and Latinx, tend to have fewer supermarkets [4, 7]. Food stores in these areas often offer fewer healthy food options [8, 9], low-quality fresh produce [10], and higher prices for the same items than in higher-income areas when healthier items are available [11]. Additionally, low-income neighborhoods tend to have more unhealthy food outlets, such as fast-food restaurants and convenience stores selling predominantly processed, energy-dense foods [12–15].

The food environment is a social determinant of health that not only influences nutrition insecurity but health outcomes [5] like cognitive function, which is critical for health and well-being in later life. Rapid cognitive decline or decline in multiple cognitive domains can be an indication of a preclinical stage of dementia [16]. Cognitive decline interferes with daily activities necessary for independent and healthy living in older adults, including driving [17], financial decision-making [18], and diabetes self-management [19]. Residing in unhealthy food environment is associated with poorer dietary quality [20–23] and food insecurity [24, 25], which are associated with accelerated cognitive decline and risk factors for dementia like obesity, diabetes, and hypertension [26–31]. Living far away from grocery stores is challenging for older adults, as they are more likely to experience dementia, diminished physical function, visual impairment, social isolation, and poverty, all of which can make accessing food stores even more difficult. However, the relationship between food environment and cognitive decline has largely been overlooked in US older adults.

The food environment encompasses various kinds of food resources, each having potentially different impacts on cognitive function, but the association of access to healthy food stores with cognitive decline in US older adults is not well understood. For example, a mixed-methods study in Minneapolis found that urban and suburban community-dwelling older adults residing in neighborhoods with more eateries (e.g., coffee shops and fast-food restaurants) had better cognitive function than their peers [32]. However, this type of food resources may confer a social benefit rather than a nutritional benefit and another study accounting for other neighborhood characteristics found opposite results [33]. A national cohort study found that residing in neighborhoods with more grocery stores was not associated with cognitive function among individuals aged 45 or older, racialized as Black and White, in metropolitan areas [33]. It is important to include both physical food access and economic conditions of the neighborhood in measuring the food environment to better capture the complex underlying contextual features. However, this measure of the food environment has not been tested in relation to cognitive decline in older adults.

To address this research gap, the current study examined the neighborhood food environments operationalized as low access to healthy food stores and low income in relation to cognitive function, leveraging data from a nationally representative sample of Medicare beneficiaries aged 65 and older living in urban communities in the US.

## Methods

### Study design and participants

**Fig. 1:**
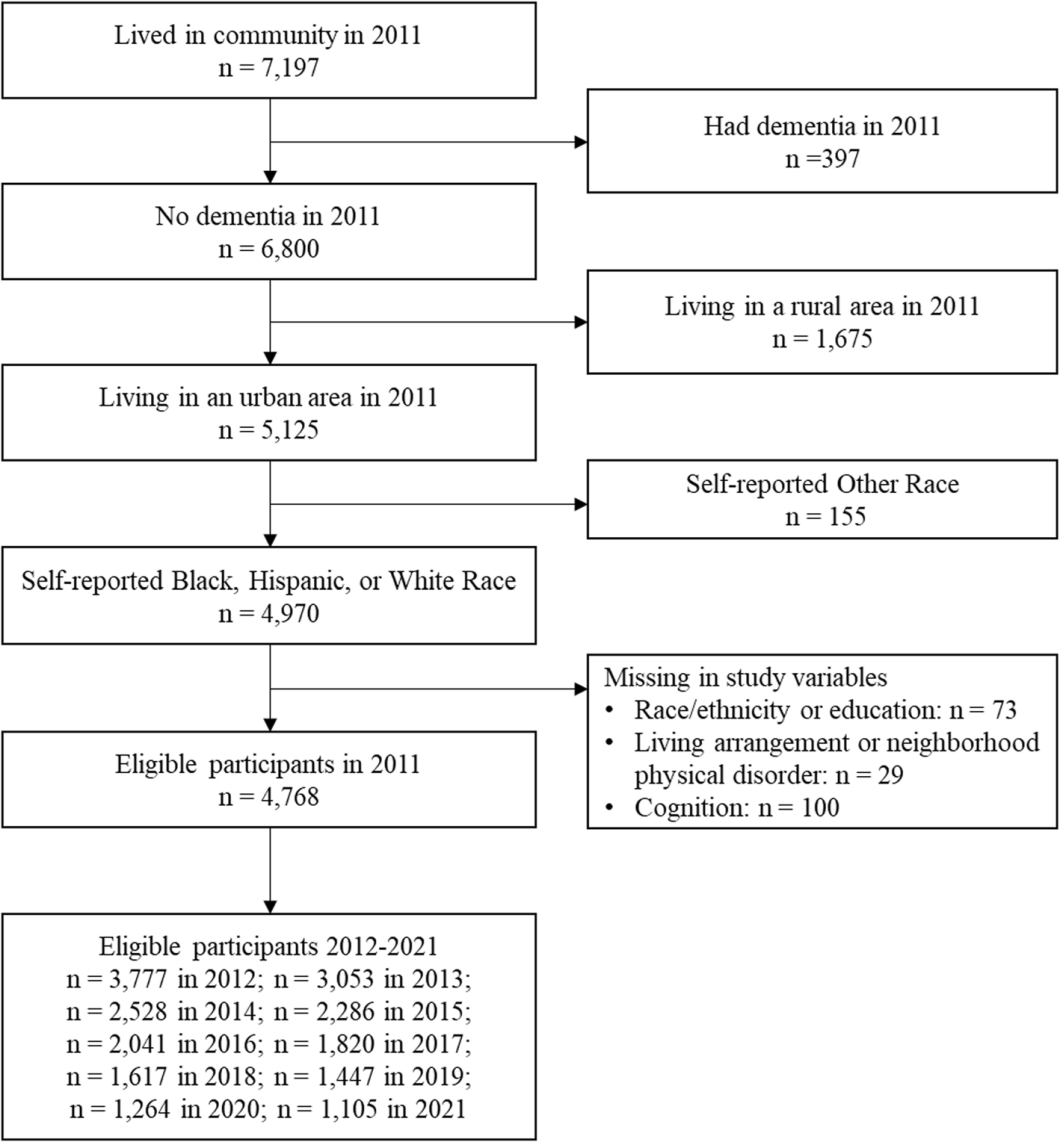
Flowchart of eligible participants over the study period (2011–2021)

This study employed a cohort study design by linking 2010 Food Access Research Atlas data [34] to the 2011–2021 National Health and Aging Trends Study (NHATS) [35]. NHATS provides data on a nationally representative cohort of Medicare beneficiaries aged 65 and older, initially recruited in 2011 and replenished in 2015 using a stratified three-stage sampling approach [36]. We included the 2011 cohort of community-dwelling participants who did not have dementia diagnosis at study enrollment and lived in urban neighborhoods (*n* = 5125). Urban neighborhoods were defined based on the US Census Bureau’s classification, where a census tract is considered urban if its geographic centroid encompasses a population of more than 2500 people [37]. NHATS conducted annual at-home interviews from 2011 to 2019, over the phone in 2020, and both in person and via phone in 2021 due to coronavirus disease of 2019 [38]. We excluded participants from rural areas since food environment in rural areas are qualitatively different from those in urban areas [39], and only few people (*n* = 91) lived in low-access and low-income neighborhoods, which limited statistical power. Additionally, fewer than 12 participants reported being racialized differently than the survey categories of Black, Latinx, or White that live in areas with low access and low income. To protect confidentiality and avoid combining the racialized groups into one group, we excluded participants who reported being racialized differently than the survey categories (*n* = 155). Lastly, we excluded participants missing study variables (*n* = 202, 4.06%) leaving a sample size of 4768 participants for this analysis (Fig. 1).

### Outcomes variables

Total cognitive function score was measured annually using domain-specific cognitive performance tests (i.e., orientation, executive function, immediate memory, and delayed memory). Orientation was assessed by querying date, month, year, and day of week and naming president and vice president (range 0 to 8). A clock drawing test was conducted to evaluate executive function, scored from 0 signifying not recognizable as a clock to 5 indicating an accurate representation of a clock [38, 40]. In 2020, materials were mailed to participants and a clock drawing test was administered over the phone. The materials were returned by mail for scoring. Immediate and delayed memory were tested by 10-word list memory and recall tests [41]. All domain-specific cognitive assessment scores were summed to create total cognitive function score (range 0 to 33), where higher scores indicate better cognitive function [42]. Previous literature suggested that there were no significant differences in total cognitive function score by survey mode [43, 44].

### Exposure variable

As in prior work, census tracts were classified as being an unhealthy food environment if they had both low food access and low income [45–47]. The Food Access Research Atlas by the US Department of Agriculture (USDA) defined low food access as a census tract where a significant number (i.e., more than 500 people or 33%) of population lived greater than 1 mile from the nearest affordable and healthy food resources including supermarkets, supercenters, or large grocery stores [37]. A census tract was considered as a low-income area if the poverty rate was greater than 20% or median family income was less than 80% of the state-wide or surrounding metropolitan area median family income [37]. Low food access and low income often co-occur in neighborhoods, creating compounded barriers to consistently and safely accessing nutritious food [48]. This overlapping of low food access and low income makes it crucial to evaluate both factors together to better predict their joint impact on health.

### Confounders

Potential confounders included individual- and area-level factors that are likely to be associated with living in area with low access and low income as well as cognitive function. These factors were measured at baseline using data from the NHATS dataset. Individual-level factors included age in year, gender (male, female), racialized group (Black, Latinx, White), educational attainment (below high school, high school graduate, above high school), log-transformed income, and living arrangement (alone, with partner, with partner and others, with others). Area-level factors included US census regions (Northeast, Midwest, South, and West) and street disorder at baseline. To assess street disorder, an interviewer rated three items from 1 (none) to 4 (a lot) and averaged the scores across items: amount of litter or broken glass on streets, graffiti on buildings, and vacant stores of houses around participants’ home [49]. Year since study enrollment (wave) was included as a time-varying variable. Street disorder was included as a confounder because low-income neighborhoods tend to have more features of disorder, such as graffiti, broken windows, and litter [50]. Additionally, neighborhood disorder has been associated with poorer cognitive function [51, 52].

### Statistical analysis

Less than 5% of observations were missing for all study variables except income (Fig. 1). The NHATS provided imputed values for total income: 13% of the sample had income values imputed within a reported bracketed value and 31% of the sample had an imputed value based on sources of income, the amount of income from each source, and auxiliary variables such as home ownership, labor force status, and educational attainment [53]. Descriptive statistics were expressed as count and percentage or mean and standard deviation and were compared for participants living in low-access and low-income neighborhoods to those not at baseline using weighted Pearson $\chi$ ^2^ statistics or bivariate linear regression model.

Mixed-effect models with random intercepts (individual) and slopes (time) were used to test the association between living in neighborhood with low access and low-income and cognitive change. Study year (wave) was used for a time metric. All models included main effect terms of study year and an indicator for living in a neighborhood with low access and low-income and their interaction term. In these models, the hypothesized association between living in neighborhood with low access and low-income and cognitive change over time was tested using the interaction term between year with low access and low-income neighborhood. The coefficient for year represented annual change in cognitive function score and the coefficient for living in a neighborhood with low access and low-income estimated the association between living in the unhealthy food environment with baseline total cognitive function score. Adjusted models controlled for baseline individual- and area-level factors. Additionally, we included interactions between time with racialized group and gender, as these factors are known to be associated with different cognitive trajectories over time [54, 55]. Analytic weights at each round were applied to account for nonresponse and the complex sampling design. Marginal means of total cognitive score over the study period were estimated from the fully adjusted mixed-effect models and used to construct a plot of total cognitive score trajectories by living in a neighborhood with low access and low-income compared to not living in these neighborhoods. Data analyses occurred between June 13, 2023 and April 30, 2024. Statistical significance was considered at *P* value of less than 0.05 (2-sided test). Analyses were conducted using Stata, version 16.1 (Stata Corp., College Station, TX).

## Results

### Participants and neighborhood characteristics

Participants included in this analysis (*N* = 4768) completed an average of 5.36 research visits (SD = 3.9). At baseline, 13.1% of participants (*n* = 717) lived in a neighborhood with low access and low-income (Table 1). The mean age of participants was 77.1 years (SD = 7.6) and 2779 were women (weighted % = 56.7). A total of 1238 participants (weighted % = 9.9%) were racialized as Black, 365 (weighted % = 9.1%) were racialized as Latinx, and 3165 (weighted % = 81.1%) were racialized as White. Participants who were racialized as Black or Latinx, as well as those with lower income and educational attainment, were more likely to live in neighborhoods with low food access and low-income. Total cognitive function score at baseline was lower among individuals living in low food access and low-income neighborhoods compared to those not living in such neighborhoods (mean = 15.7, SD = 5.4 vs. mean = 17.3, SD = 5.2, respectively).

**Table 1:** Characteristics of participants at study enrollment by neighborhood food environment, National Health and Aging Trends Study (NHATS) (*N* = 4768)

Characteristic	Not residing in a low food access or low-income neighborhood	Residing in a low food access and low-income neighborhood	*P* value
	Raw no. (weighted %)		
Overall	4051 (86.9)	717 (13.1)	
Age (year), raw mean (SD)	77.1 (7.6)	76.7 (7.7)	0.22
Gender			0.85
Male	1705 (43.3)	284 (42.9)	
Female	2346 (56.7)	433 (57.1)	
Race and ethnicity			< 0.001
Black	963 (8.9)	275 (16.8)	
Latinx	291 (8.2)	74 (14.5)	
White	2797 (82.9)	368 (68.7)	
Educational attainment			
Less than high school	933 (17.6)	278 (36.1)	< 0.001
High school	1030 (24.8)	196 (28.2)	
Above high school	2088 (57.6)	243 (35.7)	
Income			
Lowest quantile	990 (19.3)	246 (27.2)	< 0.001
2nd quantile	858 (19.1)	207 (28.8)	
3rd quantile	1048 (26.5)	168 (26.9)	
Highest quantile	1155 (35.1)	96 (17.2)	
Living arrangement			
Alone	1298 (28.7)	247 (29.5)	0.10
With spouse/partner	1682 (48.4)	255 (43.0)	
With spouse/partner and others	374 (9.5)	70 (11.3)	
With others only	697 (13.5)	145 (16.2)	
Census region			0.02
Northeast	895 (22.5)	56 (8.2)	
Midwest	858 (20.3)	172 (24.4)	
South	1475 (35.4)	371 (47.5)	
West	823 (21.8)	118 (19.9)	
Street disorder, raw mean (SD)	1.09 (0.29)	1.15 (0.37)	< 0.001
Cognitive function, raw mean (SD)	17.3 (5.2)	15.7 (5.4)	< 0.001

Weighted percentages were calculated using 2011 sampling weight to obtain estimates representing the population of Medicare beneficiaries aged 65 years and older living in urban areas

Living in neighborhoods with both low food access and low income was also associated with residing on streets with higher street disorder scores and with living in the Midwest and South census regions (Table 1). Additionally, low-income neighborhoods were more likely to be classified as low food access neighborhoods (Additional file 1: Table 1).

### Living in neighborhoods with low food access and low income and cognitive change

**Fig. 2:**
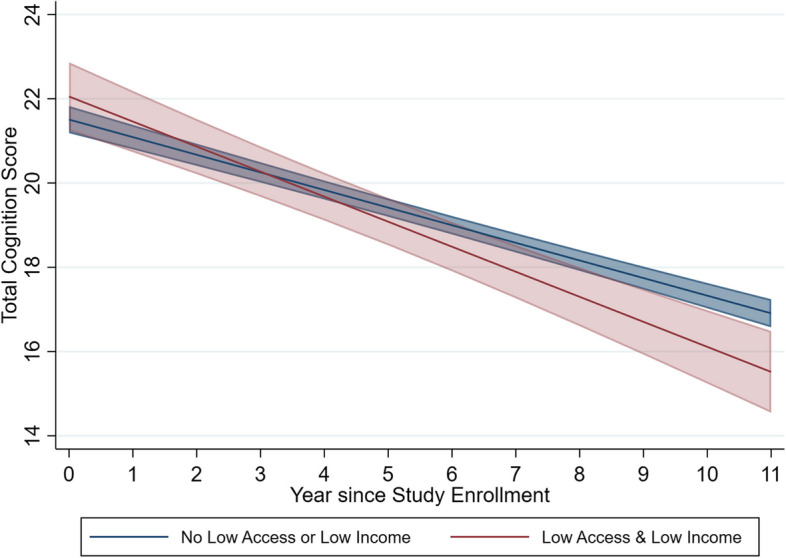
Marginal predicted mean of total cognition over the study period comparing participants residing in neighborhoods with low food access and low income to those not living in those neighborhoods

Living in a neighborhood with low access and low-income was not associated with baseline total cognitive function score in fully adjusted model (*β* = 0.58; 95% CI = − 0.35, 1.50) (Table 2). However, residing in a neighborhood with low access and low-income was associated with a faster decline in total cognitive scores (*β* = − 0.19; 95% CI = − 0.32, − 0.05). As shown in Fig. 2, the baseline total cognitive function score was slightly higher among individuals not living in low-access and low-income neighborhoods compared to their peers. However, those residing in such neighborhoods experienced a faster decline in total cognitive function over time, leading to a crossover in the cognitive function score trajectories of the two groups.

**Table 2:** Association between living in neighborhoods with low food access and low income and cognitive function among US urban community-dwelling older adults (*N* = 4768)

Model terms	Age adjusted *B* (95% CI)	*P* value	Fully adjusted *B* (95% CI)	*P* value
Low food access and low income	− 0.64 (− 1.69, 0.41)	0.23	0.58 (− 0.35, 1.50)	0.22
Study year	− 0.42 (− 0.47, − 0.36)	< 0.001	− 0.39 (− 0.46, − 0.33)	< 0.001
Low food access and low income × study year	− 0.18 (− 0.34, − 0.02)	0.03	− 0.19 (− 0.32, − 0.05)	0.01

Age adjusted model included age (year) at baseline. Fully adjusted models included baseline age (year), gender, race and ethnicity, educational attainment, log-transformed income, living arrangement, study year, census region, and street disorder, as well as interaction terms between study year with gender and race and ethnicity

The coefficient for low food access and low-income reflects the association between living in a low access and low-income neighborhood and baseline cognitive function

The coefficient for study year represents annual change in cognitive function

The interaction term between residing in a neighborhood with low food access and low-income and study year tests the study’s primary aim: the association between living in neighborhood with low access and low-income and cognitive change over time

We conducted an additional analysis to test the additive effects by creating a categorical variable. The reference group (value = 0) includes participants living in neighborhoods that were neither low-income nor low food access. A value of 1 represents living in a low-income neighborhood but not a low food access neighborhood. A value of 2 indicates living in a low food access neighborhood but not a low-income neighborhood. A value of 3 represents living in a neighborhood classified as both low-income and low food access. The findings were consistent with the results above: individuals residing in low access and low-income neighborhoods experienced a faster decline in total cognitive function compared to those living in neighborhoods that were neither low access nor low-income (*β* = − 0.19; 95% CI = − 0.35, − 0.02) (Additional file 1: Table 2).

Furthermore, we conducted a multiplicative analysis using separate binary variables for low-income and low food access. The findings were consistent with our primary analyses: residing in neighborhoods characterized by both low-income and low food access was associated with a faster decline in cognitive function over time (*β* = − 0.19; 95% CI = − 0.38, − 0.00) (Additional file 1: Table 3).

## Discussion

This study found that living in a neighborhood with low food access and low-income is associated with accelerated cognitive decline among urban community-dwelling older adults, even after adjusting for individual socioeconomic factors such as income and education. Even though we observed a small decline (0.19 points per year on a scale ranging from 0 to 33), this change is still meaningful. This is because the steeper rate of cognitive decline observed in individuals living in poor food environments, compared to those in better-resourced areas for healthy and affordable food purchasing, is not a part of normal biological aging. Rather, it likely reflects a process of accelerated cognitive decline related to structural determinants that disproportionately affect low income and racially and ethnically minoritized communities, further exacerbating existing disparities in cognitive health. This study leveraged data collected over 10 years from an ongoing nationally representative cohort of Medicare beneficiaries ages 65 and older. These findings build on prior studies by measuring urban neighborhood food environments based on having both low food access and low-income, highlighting this measure as an important indicator of health among community-dwelling older adults. This contributes to a better understanding of the interconnectedness of neighborhood lo- income status and low neighborhood food access—conditions that often overlap and co-occur—and its relationship to cognitive function among community-dwelling older adults. Furthermore, these results contribute to the literature by suggesting that the food environment is a structural risk factor for accelerated cognitive decline among older adults living in urban areas.

The association between residing in areas with low food access and low-income and cognitive decline among urban-dwelling older adults can be explained by several potential mechanisms, including poor dietary quality, food insecurity, and stress-related pathways. Limited access to healthy food is associated with poor diet quality [56] and food insecurity [57], both of which have been linked to cognitive decline [31, 58–60]. The imposed poor or insufficient diets can also contribute to greater risk for diet-related diseases such as obesity, diabetes, and hypertension [61, 62], which are risk factors for cognitive decline [63–65]. Additionally, stress or depression from living in a disadvantaged neighborhood [66] may contribute to cognitive decline through systemic inflammation, cardiovascular dysfunction, and endocrine-metabolic imbalance, referred to as allostatic load [67, 68].

Our study extends the limited body of evidence on the relationship between food environments and cognitive function in US older adults. A previous longitudinal study found inconsistent findings, suggesting that residing a neighborhood with more grocery stores (number per 1000 population in a census tract) was not associated with cognitive function score based on five cognitive tests among 221,151 Black and White Americans aged 45 or older residing in metropolitan areas [33]. The discrepancy may come from differences in sample characteristics, such as age distribution and racialized group composition because the current study was conducted in a representative sample of Medicare beneficiaries aged 65 and older. Importantly, the measure of the food environment differs: the previous study used grocery store density within a census tract, while our current study assessed low food access in terms of proximity and its combination with low-income within a census tract. Accounting for both distance to healthy food stores and the neighborhood income level may better capture the risk factors of cognitive decline that older adults face in daily lives in urban neighborhoods. This also implies the importance of considering not only access to healthy food outlets but also other contextual factors to improve healthy eating and cognitive function. Addressing only one of these factors might not be enough.

Individuals with low-income and those racialized as Black or Latinx often experience more modifiable risk factors for cognitive decline, such as physical inactivity [69, 70]. Our analysis showed that these individuals are more likely to live in neighborhoods with both low food access and low- income. Although individual factors are strong predictors of cognitive decline, structural factor—low food access and low-income neighborhoods—is independently associated with accelerated cognitive decline, even after controlling for individual-level sociodemographic factors such as income and educational attainment. Living in a neighborhood with both poor food access and low-income may create a double disadvantage by compounding individual risk factors and exacerbating disparities in diet quality, food insecurity, and cognitive decline, particularly along the lines of income, race, and ethnicity. For example, low-income households living in a neighborhood with both low access and low-income may face additional, yet avoidable, structural barriers, such as increased travel time to obtain affordable and healthy food—further burdening those already experiencing financial hardship on a limited budget [71, 72]. Addressing only individual factors may not be sufficient to reduce nutrition inequities and disparities in cognitive decline.

The association between residing in neighborhoods with both low food access and low-income and cognitive decline underscores the need to consider environmental factors to improve cognitive health outcomes for community-dwelling older adults in urban areas. For example, individual-level dietary and behavioral interventions that encourage healthy food purchasing and consumption should also assess and address participants’ food environments including proximity to healthy food stores, availability of affordable healthy food options, and access to transportation to these food outlets. Additionally, community gardens which increase access to fruits and vegetables, physical activity, and social connections [73] can be potential, environmental-level strategies in urban low food access and low-income areas. Lastly, participation in the Supplemental Nutrition Assistance Program (SNAP) has been associated with slower cognitive decline compared to non-participants [74, 75]. Policies that require SNAP retailers to stock a variety of food choices could also help increase access to healthy food options in low-income communities, thereby improving cognitive function.

This study has several limitations that should be considered when interpreting the findings. First, it is possible that people living in low food access and low-income neighborhoods at baseline may move to other locations and this can result in misclassification of exposure status. Moreover, even if individuals remain in the same location, the food environment may change over time. We explored if living in neighborhoods with both low access and low-income was associated with residential mobility during study years in our sample; adding residential mobility into the final models did not alter the inference for the hypothesized association. We also found that racially and ethnically minoritized individuals, as well as those with lower income and lower educational attainment, are more likely to reside in low food access and low-income areas. This is especially relevant for older adults, who often rely on fixed income sources. As a result, even when they relocate, they are likely to move to neighborhoods with similar income levels. These patterns may not simply reflect personal choice but rather constrained or forced decisions shaped by structural factors. Given this, we expect that these individuals may have lived in such neighborhoods not only at baseline but also in the past or during the follow-up period. As a result, the true association may be stronger when capturing the cumulative impacts of residing in low food access and low-income area, rather than assessing exposure only at baseline. Future studies should consider capturing the duration of living in low food access and low-income neighborhoods in relation to cognitive decline to better understand its accumulative impacts. Second, food purchasing and dietary habits are affected by multiple factors such as physical and social environmental characteristics, culture, and religion [76, 77], but in this study, we captured only part of food environments. Future studies should consider dynamic food systems consisting of multiple factors that affect people’s purchasing and dietary behaviors, such as food prices, opening hours, reliable transportation to food stores, social capital, neighborhood safety, culturally tailored mobile markets in multiethnic communities, and food banks to better quantify impact of food environments on healthy diets and cognitive decline. Third, despite adjusting for both individual- and neighborhood-level potential confounders, residual confounding due to unmeasured factors such as wealth and employment cannot be ruled out. However, we controlled for available socioeconomic factors such as education and income, which are strong predictors of other unmeasured individual- and neighborhood-level confounders or mediators that may be involved in causal pathways. Therefore, our estimated association is likely underestimated toward the null. Lastly, although NHATS oversampled Black individuals and we applied survey weights to enhance generalizability, Medicare beneficiaries may still not be fully representative of the US older adult population, particularly in terms of income. However, NHATS data closely align with US Census Bureau data in terms of age groups and sex when survey weights are applied [36].

## Conclusions

This study provides new knowledge highlighting the importance of neighborhood environments in which people live their daily lives for cognitive decline. The findings suggest that residing in a neighborhood with low food access and low-income is associated with an accelerated cognitive decline among urban-dwelling older adults. These findings are notable because people who are Black, Indigenous, and Latinx are likely to live in these unhealthy food environments [7, 78] and the negative association of the food environments with cognitive declines can function as additional environmental hazards for the people who are experiencing health disparities and contribute to widening the health disparities. The study findings can be helpful to develop strategies to reduce food access inequities and cognitive health disparities. Policy makers or advocates can consider increasing food access and reducing poverty as top-down intervention strategies to improve cognitive health among older populations, particularly those living in urban areas.

## Supplementary Information

Additional file 1: Tables 1–3. Table 1 Association between low-income neighborhoods and low food access neighborhoods. Table 2 Association between living in low food access and low-income neighborhoods and cognitive function: additive effects model. Table 3 Association between living in low food access and low-income neighborhoods and cognitive function: multiplicative effects model.

## Data Availability

Food Access Research Atlas data (https://www.ers.usda.gov/data-products/food-access-research-atlas/download-the-data/) and National Health and Aging Trends Study (NHATS) data (https://www.nhats.org/researcher/nhats) are publicly available. However, the Census tract codes for NHATS participants are restricted data and cannot be publicly shared.

## Abbreviations

US: United States
NHATS: National Health and Aging Trends Study
USDA: United States Department of Agriculture
SNAP: Supplemental Nutrition Assistance Program
SD: Standard deviation
CI: Confidence interval
