# Supplementary material for: Disparities in neighborhood food environment and cognitive decline among US older adults: a cohort study
Source: BMC Med. 2025 May 6;23:259. doi: 10.1186/s12916-025-04091-1 (PMC12054326; doi:10.1186/s12916-025-04091-1)
Supplement: Supplementary file 1 — Additional file 1: Tables 1–3. Table 1 Association between low-income neighborhoods and low food access neighborhoods. Table 2 Association between living in low food access and low-income neighborhoods and cognitive function: additive effects model. Table 3 Association between living in low food access and low-income neighborhoods and cognitive function: multiplicative effects model. [file 12916_2025_4091_MOESM1_ESM.docx]

**Supplementary materials for:**

**Disparities in Neighborhood Food Environment and Cognitive Decline among U.S. Older Adults: A Cohort Study**

Boeun Kim, PhD, MPH, RN^1*^; Roland J. Thorpe, Jr., PhD, MS^2^; Sarah L. Szanton, PhD, RN^2,3,4^; Paris B. Adkins-Jackson, PhD, MPH^5^; Laura J. Samuel, PhD, MSN^3^

^1^ College of Nursing, University of Iowa, 50 Newton Road, Iowa City, IA 52242, USA

^2^ Bloomberg School of Public Health, Johns Hopkins University, 624 N. Broadway, Hampton House 708, Baltimore, MD 21205, USA

^3^ School of Nursing, Johns Hopkins University, 525 N. Wolfe Street, Baltimore, MD 21205, USA

^4^ School of Medicine, Johns Hopkins University, 301 Mason F. Lord Drive, Suite 2500, Baltimore, MD 21224, USA

^5^ Mailman School of Public Health, Columbia University, 722 West 168th Street, New York, NY 10032, USA

*Correspondence: boeun-kim@uiowa.edu

**Table 1.** Association between low-income neighborhoods and low food access neighborhoods (N = 4768)

|  | | Low food access, count (%) | | Total |
| --- | --- | --- | --- | --- |
|  |  | No | Yes |  |
| Low-income | No | 1166 (24.5) | 1647 (34.5) | 2813 |
|  | Yes | 1238 (26.0) | 717 (15.0) | 1955 |
| Total | | 2404 | 2364 | 4768 |

Numbers represent the count of participants living in these neighborhoods at baseline. Overall percentages are shown, calculated based on the total sample. The *p*-value is based on the Chi-Squared test and is <0.001.

A low food access neighborhood is defined as a census tract where a significant number of residents (i.e., more than 500 people or at least 33% of the population) live more than one mile from the nearest source of affordable and healthy food, including supermarkets, supercenters, or large grocery stores. A census tract is classified as a low-income neighborhood if the poverty rate exceeds 20% or if the median family income is less than 80% of the state-wide or surrounding metropolitan area median family income.

**Table 2**. Association between living in low food access and low-income neighborhoods and cognitive function among U.S. urban community-dwelling older adults: Additive effects model (N = 4768)

| Model Terms | Age adjusted  B (95% CI) | *P* value | Fully adjusted  B (95% CI) | *P* value |
| --- | --- | --- | --- | --- |
| No low-income & no low-access (ref) |  |  |  |  |
| Low-income & no low-access | -1.76  (-2.62, -0.91) | <0.001 | 0.14  (-0.74, 1.01) | 0.76 |
| No low-income & low-access | 0.41  (-0.43, 1.26) | 0.33 | 0.31  (-0.53, 1.16) | 0.46 |
| Low-income & low-access | -0.82  (-2.08, 0.43) | 0.20 | 0.76  (-0.36, 1.88) | 0.18 |
| Study year | -0.42  (-0.51, -0.32) | <0.001 | -0.39  (-0.50, -0.29) | <0.001 |
| No low-income & no low-access x Study year (ref) |  |  |  |  |
| Low-income & no low-access x Study year | 0.01  (-0.12, 0.15) | 0.86 | -0.001  (-0.14, 0.14) | 0.99 |
| No low-income & low-access x Study year | 0.002  (-0.11, 0.12) | 0.98 | 0.001  (-0.12, 0.12) | 0.99 |
| Low-income & low-access x Study year | -0.18  (-0.37, 0.01) | 0.06 | -0.19  (-0.35, -0.02) | 0.025 |

A categorical variable was created to test the additive effects. The reference group (value = 0) includes living in neighborhoods that were neither low-income nor low food access. A value of 1 represents living in a low-income neighborhood but not a low food access neighborhood. A value of 2 indicates living in a low food access neighborhood but not a low-income neighborhood. A value of 3 represents living in a neighborhood classified as both low-income and low food access.

Age adjusted model included age (year) at baseline. Fully Adjusted models included baseline age (year), gender, race and ethnicity, educational attainment, log-transformed income, living arrangement, study year, census region, and street disorder, as well as interaction terms between study year with gender and race and ethnicity.

The coefficients for the main effects of categorized food environments reflect the association between living in each neighborhood type and baseline cognitive function, compared to living in neighborhoods that are neither low-income nor low food access (reference group). The coefficient for study year represents the annual change in cognitive function. The interaction term between categorized food environments and year examines whether the rate of cognitive change over time differs by neighborhood type, compared to the reference group (neither low-income nor low food access).

**Table 3**. Association between living in low food access and low-income neighborhoods and cognitive function among U.S. urban community-dwelling older adults: Multiplicative effects model (N = 4768)

| Model Terms | Age adjusted  B (95% CI) | *P* value | Fully adjusted  B (95% CI) | *P* value |
| --- | --- | --- | --- | --- |
| Low-income | -1.76 (-2.61, -0.92) | <0.001 | 0.14 (-0.74, 1.01) | 0.76 |
| Low-access | 0.41 (-0.43, 1.26) | 0.33 | 0.31 (-0.53, 1.16) | 0.46 |
| Low-income x low-access | 0.53 (-0.75, 1.81) | 0.41 | 0.31 (-0.92, 1.54) | 0.62 |
| Study year | -0.42 (-0.51, -0.32) | <0.001 | -0.39 (-0.50, -0.29) | <0.001 |
| Low-income x study year | 0.01 (-0.13, 0.15) | 0.86 | -0.001 (-0.14, 0.14) | 0.99 |
| Low-access x study year | 0.002 (-0.11, 0.12) | 0.98 | 0.001 (-0.12, 0.12) | 0.99 |
| Low-income x low-access x study year | -0.19 (-0.40, 0.01) | 0.07 | -0.19 (-0.38, 0.00) | 0.05 |

The coefficient of the three-way interaction term (low-income × low-access × study year) tests the multiplicative association between living in a low-income, low-access area and changes in cognitive function over time.

Age adjusted model included age (year) at baseline. Fully Adjusted models included baseline age (year), gender, race and ethnicity, educational attainment, log-transformed income, living arrangement, study year, census region, and street disorder, as well as interaction terms between study year with gender and race and ethnicity.

The coefficients for the main effects of categorized food environments reflect the association between living in each neighborhood type and baseline cognitive function, compared to living in neighborhoods that are neither low-income nor low food access (reference group). The coefficient for study year represents the annual change in cognitive function. The interaction term between categorized food environments and year examines whether the rate of cognitive change over time differs by neighborhood type, compared to the reference group (neither low-income nor low food access).
